# Forces to Drive Neuronal Migration Steps

**DOI:** 10.3389/fcell.2020.00863

**Published:** 2020-09-01

**Authors:** Takunori Minegishi, Naoyuki Inagaki

**Affiliations:** Laboratory of Systems Neurobiology and Medicine, Division of Biological Science, Nara Institute of Science and Technology, Nara, Japan

**Keywords:** neuronal migration, mechanobiology, traction force, adhesion force, mechanical tension, shootin1, actomyosin, dynein

## Abstract

To establish and maintain proper brain architecture and elaborate neural networks, neurons undergo massive migration. As a unique feature of their migration, neurons move in a saltatory manner by repeating two distinct steps: extension of the leading process and translocation of the cell body. Neurons must therefore generate forces to extend the leading process as well as to translocate the cell body. In addition, neurons need to switch these forces alternately in order to orchestrate their saltatory movement. Recent studies with mechanobiological analyses, including traction force microscopy, cell detachment analyses, live-cell imaging, and loss-of-function analyses, have begun to reveal the forces required for these steps and the molecular mechanics underlying them. Spatiotemporally organized forces produced between cells and their extracellular environment, as well as forces produced within cells, play pivotal roles to drive these neuronal migration steps. Traction force produced by the leading process growth cone extends the leading processes. On the other hand, mechanical tension of the leading process, together with reduction in the adhesion force at the rear and the forces to drive nucleokinesis, translocates the cell body. Traction forces are generated by mechanical coupling between actin filament retrograde flow and the extracellular environment through clutch and adhesion molecules. Forces generated by actomyosin and dynein contribute to the nucleokinesis. In addition to the forces generated in cell-intrinsic manners, external forces provided by neighboring migratory cells coordinate cell movement during collective migration. Here, we review our current understanding of the forces that drive neuronal migration steps and describe the molecular machineries that generate these forces for neuronal migration.

## Introduction

Neuronal migration is a fundamental process to establish and maintain the nervous system (Hatten, 2002; Ayala et al., 2007; Ghashghaei et al., 2007; Marin et al., 2010; Kaneko et al., 2017), and defects in neuronal migration cause a number of disorders including brain malformation, intellectual disability, epilepsy and psychiatric diseases (Valiente and Marin, 2010; Evsyukova et al., 2013; Moffat et al., 2015; Stouffer et al., 2016). Decades of intensive analyses of mouse mutants and human brain malformations have yielded substantial progress in our understanding of the molecular bases for controlling neuronal migration (Hatten, 2002; Govek et al., 2011; Hirota and Nakajima, 2017). In addition, imaging analyses have uncovered spatiotemporal molecular and cellular events underlying neuronal migration (Famulski et al., 2010; Yanagida et al., 2012; Cooper, 2013; Hatanaka et al., 2016; Ohtaka-Maruyama et al., 2018; Saito et al., 2019). Although these studies have significantly advanced our understanding of neuronal migration on the molecular and cellular levels, cell movement ultimately depends on the generation of driving forces. Therefore, one of the major goals of current research is understanding the molecular machineries required to generate forces for neuronal migration.

**FIGURE 1:**
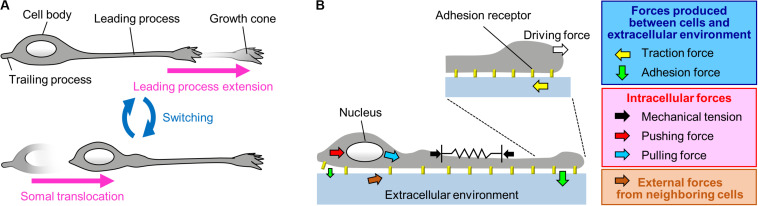
A Mechanical model for neuronal migration. **(A)** Neurons migrate in a saltatory manner by repeating the two distinct steps: leading process extension and somal translocation. **(B)** The driving force for leading process extension (white arrow) is produced as a counter force of the traction force on the adhesive substrate produced by the growth cone (yellow arrow). Somal translocation is likely to be driven by multiple forces, including mechanical tension along the leading process (black arrows), a decrease in the adhesion force at the cell body (smaller green arrow), and pushing (red arrow) and pulling (blue arrow) forces exerted on the nucleus. In addition, forces provided by neighboring cells (brown arrow) coordinate cell movement during collective migration.

Migrating neurons exhibit bipolar morphology, with a long leading process and a short trailing process (Tsai and Gleeson, 2005; Marin et al., 2006); the tip of the leading process bears a highly motile structure, the growth cone (Marin et al., 2006; Ayala et al., 2007; Marin et al., 2010; Cooper, 2013; Evsyukova et al., 2013; Figure 1A). Typically, neurons migrate in a saltatory manner by repeating two distinct steps, namely extension of the leading process and translocation of the cell body (Edmondson and Hatten, 1987; O’Rourke et al., 1992; Komuro and Rakic, 1993, 1995; Wichterle et al., 1997; Nadarajah et al., 2001, 2002; Schaar and McConnell, 2005; Tsai and Gleeson, 2005; Marin et al., 2006; Figure 1A). Neurons must therefore generate forces to extend the leading process as well as to translocate the cell body. In addition, they need to switch these forces alternately in order to orchestrate their saltatory movement. This review outlines recent findings and mechanobiological approaches that are beginning to uncover the forces required to drive neuronal migration. Spatiotemporally organized forces produced between neurons and the extracellular environment play key roles in driving these migration steps (blue box, Figure 1B). Namely, the driving force for leading process extension (white arrow, Figure 1B) is produced as a counter force of the traction force on the environment (yellow arrow, Figure 1B) generated by the growth cone. On the other hand, a decrease in the adhesion force at the cell body (smaller green arrow, Figure 1B) propels somal translocation. In addition, intracellular forces (red box, Figure 1B), including leading process tension (black arrows, Figure 1B) and pushing and pulling forces exerted on the nucleus (red and blue arrows, Figure 1B), contribute to somal translocation. Furthermore, forces provided by neighboring migratory cells (brown arrow, Figure 1B) coordinate cellular movement during collective migration. Concerning the mechanical regulation of nuclear translocation, readers are also referred to other reviews (Tsai and Gleeson, 2005; Marin et al., 2010; Trivedi and Solecki, 2011; Nakazawa and Kengaku, 2020).

## Forces for Leading Process Extension

### Traction Force at the Growth Cone Extends the Leading Process

**FIGURE 2:**
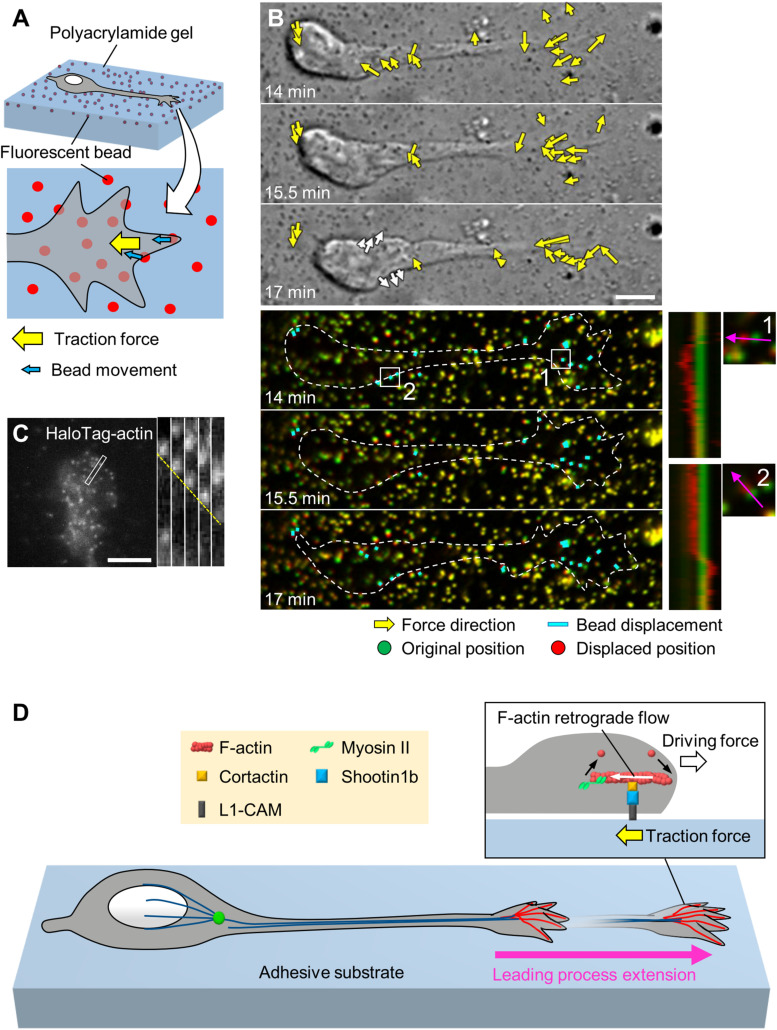
Generation of traction force for leading process extension by actin–adhesion coupling. **(A)** Schema of traction force microscopy to monitor force generated by migrating neurons. Neurons are cultured on polyacrylamide gel coated with adhesive substrates such as L1-CAM; fluorescent beads are embedded in the gel. Traction forces under the cell (yellow arrow) are monitored by visualizing force-induced deformation of the gel, which is reflected by the movement of beads under the neuron (blue arrows). **(B)** Force mapping of a migrating neuron. Differential interference contrast (DIC, upper panels) and fluorescence (lower panels) time-lapse images of a migrating olfactory interneuron (see Supplementary Video S1). The original and displaced positions of the beads are indicated by green and red, respectively, while the bead displacements are indicated by cyan rectangles. Yellow arrows in DIC images indicate the magnitude and direction of traction forces. Dashed lines indicate the boundary of the cell. The kymographs (lower right) along the axis of bead displacement (pink arrows) at the boxed areas 1 and 2 of the neuron show movement of beads. Note that the gel under the cell body deformed forward during the somal translocation step (white arrows in the bottom DIC image and box 2). Modified from Minegishi et al. (2018) (This work is licensed under the CC BY license, https://creativecommons.org/licenses/by/4.0/) with permission. **(C)** Fluorescent speckle image of HaloTag-actin at the leading process growth cone of an olfactory interneuron, and kymograph of the boxed area at 3 s intervals (right) (see Supplementary Video S2). The dashed line indicates the retrograde flow of speckles. Reproduced from Minegishi et al. (2018) with permission. **(D)** Molecular machinery for generation of traction force in migrating olfactory interneurons. At the leading process growth cone, shootin1b mediates actin–adhesion coupling, through its interactions with cortactin and L1-CAM. This coupling generates traction force under the growth cone (yellow arrow). The driving force for leading process extension (forward white arrow) is generated as a counterforce to the traction forces exerted on the adhesive substrate. Scale bars: 5 μm.

Neurons migrate within tightly packed environments, including glial cells, other neurons and the extracellular matrix (ECM) (Franco and Muller, 2011; Solecki, 2012), which serve as adhesive substrates. To migrate through these environments, neurons need to produce forces against the adhesive substrates. Indeed, using traction force microscopy (Figure 2A), recent studies detected traction forces produced by migrating cerebellar granule cells in 2D conditions (Jiang et al., 2015; Umeshima et al., 2019) and by olfactory interneurons in a semi-3D condition (Minegishi et al., 2018). In all cases, prominent traction forces were observed at the growth cone of the leading process (yellow arrows, Figure 2B and Supplementary Video S1). The direction of the traction forces at the growth cone was oriented toward the rear of the cell (Minegishi et al., 2018; Umeshima et al., 2019). In addition, the magnitude of the forces showed a positive correlation with the speed of growth cone advance (Minegishi et al., 2018), indicating that the traction forces generated at the growth cone drive leading process extension.

### Shootin1b Mediates Actin–Adhesion Coupling for Generation of Traction Force at the Leading Process Growth Cones

The tip of an extending axon also bears a growth cone (Lowery and Van Vactor, 2009), and axonal growth cones produce traction forces for axon outgrowth and guidance (Chan and Odde, 2008; Koch et al., 2012; Abe et al., 2018; Baba et al., 2018). Decades of analyses of axonal growth cones have revealed a key machinery to generate traction forces for growth cone migration. At the leading edge of the axonal growth cone, actin filaments (F-actins) polymerize and disassemble proximally, which, in conjunction with myosin II activity, induces retrograde flow of F-actins (Forscher and Smith, 1988; Katoh et al., 1999; Medeiros et al., 2006). Mechanical coupling between F-actin retrograde flow and adhesive substrates through clutch and adhesion molecules generates traction forces on the substrates (Mitchison and Kirschner, 1988; Suter and Forscher, 2000; Toriyama et al., 2013). Namely, the actin–adhesion coupling transmits the force of F-actin retrograde flow to the adhesive substrate, producing traction force on the substrate. Concurrently, actin–adhesion coupling reduces the speed of the F-actin retrograde flow, thereby converting actin polymerization into force that pushes the leading-edge membrane. To date, shootin1a is one of the best-characterized clutch molecules involved in the generation of traction forces at the axonal growth cone (Toriyama et al., 2006; Shimada et al., 2008). Shootin1a interacts with F-actin retrograde flow through its association with the F-actin-interacting protein cortactin (Kubo et al., 2015). Shootin1a also interacts with the cell adhesion molecule L1-CAM (Baba et al., 2018), which binds to the ECM protein laminin (Abe et al., 2018) as well as to L1-CAM expressed on neighboring cells (Lemmon et al., 1989), thereby mechanically coupling the F-actin retrograde flow with the adhesive substrates. The shootin1a-mediated actin–adhesion coupling generates traction forces for axon outgrowth (Kubo et al., 2015) and axon guidance induced by diffusible and substrate-bound chemical cues (Abe et al., 2018; Baba et al., 2018). N-cadherin–catenin complexes were also reported to mediate actin–adhesion coupling at the axonal growth cone (Bard et al., 2008; Garcia et al., 2015).

As in the case of axonal growth cones, F-actins also undergo retrograde flow at the tip of leading process growth cones (Figure 2C and Supplementary Video S2; He et al., 2010; Minegishi et al., 2018). A recent study reported that shootin1b, a splicing variant of shootin1a (Higashiguchi et al., 2016), functions as a clutch molecule at the leading process growth cone of migrating olfactory interneurons (Minegishi et al., 2018; Figure 2D). During neuronal migration, shootin1b undergoes dynamic accumulation in the leading process growth cone; this accumulation positively correlates with leading process extension. Shootin1b at the growth cone couples F-actin retrograde flow and cell adhesions via cortactin and L1-CAM, thereby generating traction force on the adhesive substrate (Minegishi et al., 2018; yellow arrow, Figure 2D). In addition, a recent study reported that shootin1b directly interacts with F-actin and promotes actin polymerization *in vitro* (Zhang et al., 2019). The driving force for leading process extension (forward white arrow) is produced as a counter force of the traction force. Shootin1 knockout (KO) decreased the magnitude of the traction force produced by the growth cone and reduced the extension of the leading process as well as the speed of neuronal migration. Furthermore, shootin1 KO led to abnormal positioning of olfactory interneurons and dysgenesis of the olfactory bulb (Minegishi et al., 2018). These data indicate that traction force generated by shootin1b-mediated actin–adhesion coupling promotes leading process extension for migration of olfactory interneurons.

In addition, shootin1 KO results in ectopic accumulation of mitral cells (Minegishi et al., 2018), olfactory excitatory neurons that undergo radial migration (Hinds, 1968; Blanchart et al., 2006). Recent studies also reported that shootin1 knockdown inhibits the radial migration of cortical neurons (Sapir et al., 2013) and that shootin1 KO delays the collective cell migration of zebrafish posterior lateral line primordium (PLLP), a cluster of progenitor cells destined to form a mechanosensory organ called the neuromast (Urasaki et al., 2019). These data suggest that traction force generated by shootin1-mediated actin–adhesion coupling may propel the migration of multiple types of neurons.

## Forces for Somal Translocation

### Tension Along the Leading Process for Somal Translocation

**FIGURE 3:**
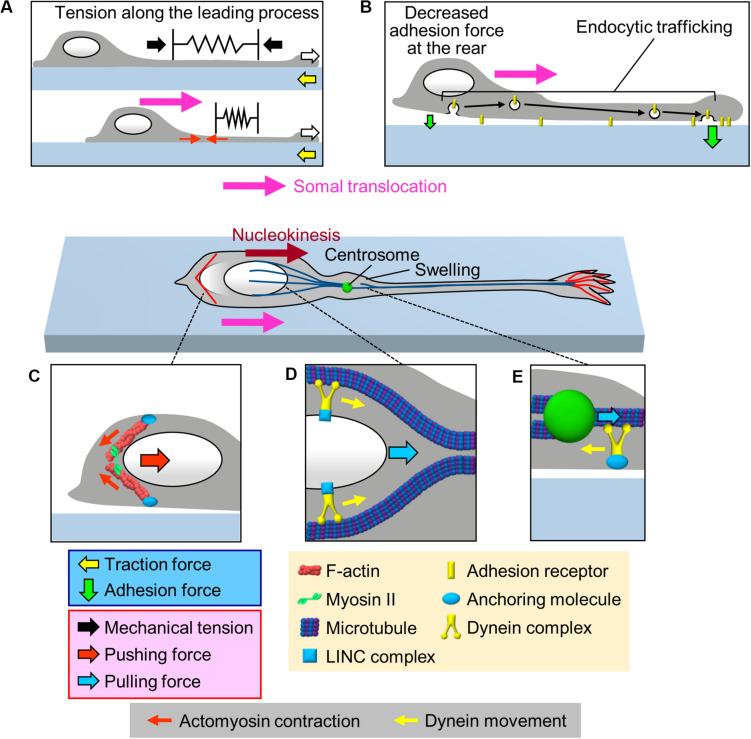
Multiple forces that cooperate for somal translocation. **(A)** Leading process extension (white arrows) increases the mechanical tension along the leading process (black arrows), which in turn pulls the cell body for somal translocation. In addition, actomyosin contraction at the proximal region of the leading process (red arrows) increases the tension along the leading process. **(B)** Decrease in the adhesion force at the cell body (smaller green arrow) propels somal translocation. Adhesion receptors are transported from the cell body to the leading process via endocytic pathways (black arrows), resulting in an increase in the adhesion force at the leading process and a decrease in adhesion force at the cell body (smaller green arrow). **(C)** Actomyosin, which may be anchored to the cell cortex, contracts at the rear of the nucleus (small red arrows), thereby squeezing the nucleus and generating pushing force (large red arrow) for nucleokinesis. **(D)** The dynein complex mechanically interacts with the nucleus via the LINC complex, and its movement (yellow arrows) along perinuclear microtubules generates pulling force (blue arrow) for nucleokinesis. **(E)** In the swelling of the proximal part of the leading process, the dynein motor complex may be immobilized on a cellular component via an anchoring molecule. The force of dynein movement (yellow arrow) slides microtubules forward, thereby pulling (blue arrow) the centrosome forward.

The cell body is the swollen part of migrating neurons; therefore, its translocation against the mechanical barriers of the surrounding environment must rely on the generation of robust forces. One of the candidate forces for mediating somal translocation is tension along the leading process (Figure 3A). He et al. (2010) reported that severing the leading process of cerebellar granule cells arrested somal translocation, demonstrating that the leading process is required for somal translocation. Consistently, in cerebellar granule cells and gonadotropin-releasing hormone (GnRH)-expressing neurons, F-actins located along the leading process move forward in correlation with somal translocation (Solecki et al., 2009; He et al., 2010; Hutchins et al., 2013; Hutchins and Wray, 2014). In addition, traction force microscopy demonstrated that the gel substrate under the cell body of olfactory interneurons deformed forward during the somal translocation step (Minegishi et al., 2018; white arrows and Box 2, Figure 2B and Supplementary Video S1). These data suggest that the leading process pulls the cell body for somal translocation (Solecki et al., 2009; He et al., 2010; Hutchins et al., 2013; Hutchins and Wray, 2014; Minegishi et al., 2018; Figure 3A). As described above, shootin1b promotes leading process extension of olfactory interneurons (white arrows, Figure 3A) by producing traction force at the growth cone (yellow arrows); on the other hand, shootin1b is also involved in somal translocation (Minegishi et al., 2018). A previous study with chick sensory neurons demonstrated that mechanical tension along neurites increases according to neurite extension (Lamoureux et al., 1989). Therefore, the leading process extension driven by traction force at the growth cone (Jiang et al., 2015; Minegishi et al., 2018; Umeshima et al., 2019) would increase tension along the process (black arrows, Figure 3A), which in turn pulls the cell body for somal translocation.

In the case of migrating cerebellar granule cells which extend F-actin-enriched leading process (Rivas and Hatten, 1995; Solecki et al., 2009), Myosin II and myosin light chain kinase (MLCK) accumulate at the proximal region of the leading process (Solecki et al., 2009; Umeshima et al., 2019). Consistently, traction force analyses detected a myosin II dependent contraction center at the proximal region of the leading process during somal translocation step (Jiang et al., 2015; Umeshima et al., 2019). Thus, actomyosin contraction would actively contribute to increase the tension along the leading process of cerebellar granule cells (red arrows, Figure 3A), thereby pulling the soma (Solecki et al., 2009; Trivedi and Solecki, 2011; Jiang et al., 2015; Umeshima et al., 2019). Recent studies have developed fluorescent tension probes that are applied in fluorescence lifetime imaging (FLIM) (Colom et al., 2018) and fluorescence resonance energy transfer (FRET) imaging (Li et al., 2018). Detailed analyses of mechanical tension along the leading process remains an important issue for future studies.

### Decrease in Adhesion Force at the Soma for Somal Translocation

As described above, cell adhesion is required for the generation of traction force for neuronal migration. On the other hand, it was also proposed that a decrease in the adhesion force at the rear of the migratory cells facilitates forward movement of cells (Sheetz et al., 1998). Indeed, overexpression or knockdown of cell adhesion molecules, such as N-cadherin or L1-CAM, inhibits neuronal migration (Kawauchi et al., 2010; Shikanai et al., 2011; Kishimoto et al., 2013; Tonosaki et al., 2014; Mestres et al., 2016), implying that coordinated regulation of adhesion forces generated between neurons and adhesive substrates is required for neuronal migration. To assess the spatial dynamics of adhesion force in migrating cerebellar granule cells, Jiang et al. (2015) performed a cell detachment assay. They mechanically pulled the middle region of the leading process of cerebellar granule cells using a micropipette, and examined the first detachment point of the neurons. In neurons whose soma was stationary, the growth cone was detached first from the substrate. In contrast, in neurons whose soma was moving forward, the cell body was detached first (Jiang et al., 2015). These data suggest that a decrease in the adhesion force at the cell body is important to propel somal translocation (Figure 3B). Although this assay is qualitative, other recent studies have reported a quantitative cell detachment assay: using femtosecond lasers combined with atomic force microscopy (AFM), controlled impulsive forces to induce cell detachment can be applied to estimate the adhesion force of cells (Hosokawa et al., 2011; Iino et al., 2016). In addition, cell adhesion molecules tagged with pH-sensitive GFP could enable analyses of spatiotemporal dynamics of adhesions in migrating neurons (Famulski et al., 2010). Quantification and spatiotemporal analyses of adhesion forces in migrating neurons are important issues for future research.

Several studies have reported an involvement of endocytic trafficking of cell adhesion molecules in regulation of spatiotemporal dynamics of cell adhesion during neuronal migration (Kawauchi et al., 2010; Wilson et al., 2010; Shieh et al., 2011; Mestres et al., 2016; Figure 3B). Kawauchi et al. (2010) performed loss-of-function assays and proposed that N-cadherin is internalized at the cell body by Rab5-dependent endocytic pathways and transported to the leading process by a Rab11-dependent recycling pathway; disruption of these trafficking pathways led to migration defects in cortical projection neurons. Similarly, Shieh et al. (2011) reported that inhibition of endocytosis by loss-of-function of dynamin led to an accumulation of integrin β1 at the cell rear, leading to disruption of the migrations of olfactory interneurons and cortical projection neurons. On the other hand, knockdown of the early endosomal protein SARA (Smad anchor for receptor activation), increased surface expression of L1-CAM and delayed radial migration of cortical neurons (Mestres et al., 2016). These findings suggest that endocytic trafficking (black arrows, Figure 3B) decreases the number of adhesion receptors at the cell body for somal translocation.

### Pushing Forces for Nucleokinesis Generated by Actomyosin Contraction

As somal translocation occurs in a saltatory manner, this step would not be explained simply in terms of a balance between the leading process tension and somal adhesion. Since the nucleus is the largest organelle in the cell body, its movement, nucleokinesis, is critical for somal translocation (Tsai and Gleeson, 2005). Accumulating evidence indicates that actomyosin contraction contributes to nucleokinesis by squeezing the nucleus (Figure 3C). During nucleokinesis of olfactory and medial ganglionic eminence (MGE)-derived interneurons, F-actin and myosin II accumulate at the rear of the nucleus, where myosin II is activated (Bellion et al., 2005; Schaar and McConnell, 2005; Martini and Valdeolmillos, 2010). Live imaging analyses demonstrated that the F-actin accumulation at the rear precedes nuclear movement. Furthermore, inhibition of myosin II activity by blebbistatin abolishes F-actin accumulation at the rear, thereby inhibiting nuclear squeezing as well as nuclear translocation (Martini and Valdeolmillos, 2010). These findings indicate that actomyosin at the rear squeezes the nucleus (small red arrows, Figure 3C) and exerts pushing force (large red arrow) to drive nucleokinesis in interneurons (Bellion et al., 2005; Schaar and McConnell, 2005; Martini and Valdeolmillos, 2010).

On the other hand, in the case of cerebellar granule cells, F-actins do not accumulate at the rear of the nucleus (Solecki et al., 2009; He et al., 2010; Umeshima et al., 2019). In addition, traction force analyses failed to detect pushing forces at the rear of these neurons (Jiang et al., 2015), thereby suggesting that actomyosin dynamics at the rear differs depending on the cell types (Trivedi and Solecki, 2011).

### Pulling Force for Nucleokinesis Generated by Dynein Motor Complex

Live-cell imaging analyses demonstrated that somal translocation is preceded by a swelling of the proximal part of the leading process and forward movement of the centrosome into this swelling (Bellion et al., 2005; Schaar and McConnell, 2005; Tsai and Gleeson, 2005; Marin et al., 2010; Shinohara et al., 2012). Accumulating data suggest that coupling between the centrosome and the nucleus plays an important role in somal translocation (Tsai and Gleeson, 2005; Marin et al., 2010; Cooper, 2013; Kaneko et al., 2017). In migrating neurons, the centrosome acts as a microtubule organizing center and extends microtubules to the leading process and to the nucleus; therefore, centrosome-organized microtubules are oriented with their minus end toward the centrosome (Tsai and Gleeson, 2005; Marin et al., 2010). Previous studies reported that minus-end-directed Lis1/Ndel1/dynein motor complex is responsible for both nucleokinesis and centrosomal movement (Shu et al., 2004; Tanaka et al., 2004; Tsai et al., 2007; Zhang et al., 2009). Perinuclear microtubules act as the scaffold for dynein-mediated nucleokinesis (Shu et al., 2004; Tanaka et al., 2004; Tsai et al., 2007). The dynein complex pulls the nucleus forward via the LINC complex (blue arrow, Figure 3D), which is formed by the transmembrane SUN and KASH proteins (Zhang et al., 2009). On the other hand, less is known about the molecular mechanism by which the dynein complex drives the forward movement of the centrosome. Tsai et al. (2007) proposed that dynein motor slides microtubules forward, thereby driving the centrosome movement (blue arrow, Figure 3E). This idea is supported by live-cell imaging data that demonstrate forward movement of microtubules in the proximal leading process (Rao et al., 2016). To underpin the centrosomal movement, the dynein complex must be immobilized on a cellular component; however, the molecular linkage for dynein immobilization remains unclear. Recent studies reported that actomyosin accumulates in front of the nucleus of migrating cerebellar granule cells, suggesting that actomyosin contraction may also contribute to pulling the nucleus for somal translocation of these cells (Solecki et al., 2009; Jiang et al., 2015; Umeshima et al., 2019). Further analyses are required to uncover the detailed molecular mechanics of nuclear translocation.

### Forces Externally Provided by Neighboring Cells During Collective Cell Migration

Some of neuronal progenitor cells, for example the cranial neural crest and zebrafish PLLP, migrate as cell clusters during development (Ghysen and Dambly-Chaudiere, 2007; Nogare et al., 2017; Shellard and Mayor, 2019). In addition, neonatal and adult olfactory interneurons undergo a stream-type collective cell migration called chain migration (Marin and Rubenstein, 2003; Rorth, 2009; Kaneko et al., 2017). In such cases, migrating cells receive forces from neighboring migratory cells (brown arrow, Figure 1B), and thus their migration is affected by the movements of the neighboring cells. For example, a recent study reported that contraction of “supracellular” actomyosin ring localized at the rear of the neural crest cell cluster drives collective migration of the cell group (Shellard et al., 2018). In the case of endothelial cells, it is proposed that cadherin-mediated cell-cell junctions between leader and follower cells orient cellular movement during collective migration (Hayer et al., 2016). Similarly, chain migration is thought to be associated with the efficient and coordinated movement of olfactory interneurons (Kaneko et al., 2017).

## Concluding Remarks

With the aid of emerging mechanobiological approaches, the molecular mechanics underlying neuronal migration is beginning to be elucidated. In this review, we have described a current view of the forces that drive the two neuronal migration steps, leading process extension and somal translocation. Spatiotemporally organized forces produced between neurons and the extracellular environment, as well as intracellular forces, play pivotal roles to drive these migration steps. Diverse molecules may contribute to the generation of these forces, depending on the neuronal cell type. As an important question, it remains unknown how neurons can switch between these processes. Molecular and mechanical interactions between the leading process and the cell body should coordinate the two processes to achieve saltatory movement. Ca^2+^ imaging analyses demonstrated transient increases in Ca^2+^ concentration in the cell body of cerebellar granule cells, which positively correlated with their somal translocation (Komuro and Rakic, 1996; Kumada and Komuro, 2004). In addition, treatment with BAPTA, a calcium chelator, abolished the transient Ca^2+^ increases and F-actin accumulation at the rear of the nucleus, resulting in inhibition of somal translocation (Martini and Valdeolmillos, 2010). These reports support the notion that transient Ca^2+^ increases are involved in the activation of actomyosin to trigger nucleokinesis. Similarly, shootin1b underwent dynamic accumulation at the leading process growth cone of migrating olfactory interneurons, which positively correlated with leading process extension (Minegishi et al., 2018). In addition, shootin1 KO inhibited the leading process extension, suggesting that the shootin1b accumulation triggers leading process extension (Minegishi et al., 2018). To understand how migratory neurons produce forces for their pathfinding, it is also important to link guidance cues in the extracellular environments with the regulation of the machineries involved in force generation. Such extracellular cues would include diffusible and substrate-bound chemical cues and mechanical properties of the environment. Further detailed measurement of forces, in combination with molecular and cell biological approaches, will enhance our understanding of the mechanics underlying neuronal migration.

## Author Contributions

TM and NI contributed cooperatively to the conceptual development, literature research, and writing of the manuscript. Both authors contributed to the article and approved the submitted version.

## Conflict of Interest

The authors declare that the research was conducted in the absence of any commercial or financial relationships that could be construed as a potential conflict of interest.

## Supplementary Material

VIDEO S1 A force-mapping video of a migrating neuron. This video shows DIC (upper) and fluorescence (lower) time-lapse imaging of a migrating olfactory interneuron. Arrows in the DIC image indicate strength and direction of traction forces (force magnitude is shown by the length of the arrows, which are 4.5 times longer than bead displacements). The original and displaced positions of the beads are indicated by green and red, respectively. The bead displacements are also indicated by cyan rectangles. Images were acquired every 30 s for 24.5 min. Scale bar, 5 μm. Modified from Minegishi et al. (2018) with permission (see Figure 2B).

VIDEO S2 Fluorescent speckle imaging of HaloTag-actin at the leading process growth cone of an olfactory interneuron. Images were acquired every 3 s for 42 s. Scale bar, 5 μm. Reproduced from Minegishi et al. (2018) with permission (see Figure 2C).
